# Transcriptomics profiles in intestinal sulfide overproduction, small intestinal bacterial overgrowth, and intestinal methanogen overgrowth

**DOI:** 10.1128/msystems.00458-26

**Published:** 2026-06-29

**Authors:** Juliana de Freitas Germano, Gabriela Leite, Maria Jesus Villanueva-Millan, Daniel Brimberry, Mohamad Rashid, Ava Hosseini, Dilara Flor, Said Bogatyrev, Walter Morales, Stacy Weitsman, Maritza Sanchez, Ignacio Rivera, Cristina Moreno Fajardo, Victoria Murray, Gonzalo Parodi, Margie Parra, Zhe Lyu, Gillian M. Barlow, Ali Rezaie, Ruchi Mathur, Mark Pimentel

**Affiliations:** 1 Medically Associated Science and Technology (MAST) Program, Cedars-Sinai 5149 , Los Angeles, California, USA; 2 Department of Plant and Microbial Biology, North Carolina State University 6798, Raleigh, North Carolina, USA; 3 Karsh Division of Gastroenterology and Hepatology, Department of Medicine, Cedars-Sinai 5149 , Los Angeles, California, USA; 4 Division of Endocrinology, Diabetes, and Metabolism, Department of Medicine, Cedars-Sinai 5149 , Los Angeles, California, USA; The Forsyth Institute, Cambridge, Massachusetts, USA

**Keywords:** small intestinal bacterial overgrowth, intestinal methanogen overgrowth, intestinal sulfide overproduction, transcriptomics, small bowel, hydrogen sulfide, methane, redox balance, mitochondrial function, immunoregulation

## Abstract

Despite advances in identifying distinct microbial profiles linked to intestinal sulfide overproduction (ISO), small intestinal bacterial overgrowth (SIBO), and intestinal methanogen overgrowth (IMO), differences in host small bowel (SB) gene expression remain unexplored. Therefore, we compared host gene transcriptomics in SB biopsies from subjects with IMO, ISO, and SIBO. Separately, SB transcriptomics in rats gavaged with hydrogen sulfide (H_2_S) producers were also analyzed. ISO subjects exhibited unique transcriptomics profiles. ISO (≥1.5 parts per million [ppm] H_2_S) broadly affected genes linked to H₂S toxicity, such as electron transport chain and redox balance genes, while there were fewer significant effects on gene expression in IMO and SIBO. Rat data supported ISO findings, including dysregulation of mitochondrial respiration, redox balance, water homeostasis, immune response, and gut motility.

**IMPORTANCE:**

Intestinal sulfide overproduction (ISO), intestinal methanogen overgrowth (IMO), and small intestinal bacterial overgrowth (SIBO) are conditions associated with higher levels of breath hydrogen sulfide and methane, respectively, and higher bacterial colony counts on small bowel aspirate cultures. While these conditions correlate with gastrointestinal symptoms, the cellular mechanisms involved when analyzed either separately or concomitantly are not well understood. Here, we aimed to explore changes in human small bowel transcriptomics in ISO, IMO, and/or SIBO, and to investigate if changes in ISO reflect those seen in models of rats gavaged with known hydrogen sulfide producers. The results demonstrate that each condition is unique and has its own transcriptomics profile, with ISO showing a greater effect on the host, which was partially validated by transcriptomic analyses in the small bowel of rats gavaged with hydrogen sulfide-producing bacteria. This study supports the need for individualized therapeutic strategies for each condition in the future.

## INTRODUCTION

Small intestinal bacterial overgrowth (SIBO), intestinal methanogen overgrowth (IMO), and intestinal sulfide overproduction (ISO) are conditions with distinct gut microbial imbalances (1–3). These conditions are closely associated with irritable bowel syndrome (IBS) (4), the most common gastrointestinal (GI) disorder worldwide (5). Recent studies have revealed that IBS affects 6.1% of the U.S. population, a greater prevalence than previously estimated (6). Despite its prevalence, IBS remains widely underdiagnosed, with only 3.3% of affected individuals receiving a medical diagnosis, while 10.8% meet the diagnostic criteria without a formal diagnosis (7). Moreover, the median total healthcare costs per year for IBS subjects can be twice as high as for non-IBS subjects (8).

SIBO, usually associated with *Escherichia* and *Klebsiella* overgrowth (3, 9–11), is defined by bacterial counts ≥10^3^ colony-forming units (CFU) per mL of small bowel (SB) aspirate detected by culture on MacConkey agar (3), and characterized by symptoms such as diarrhea, bloating, and abdominal pain (9). Alternatively, SIBO can be diagnosed by hydrogen (H_2_) breath test levels ≥20 parts per million (ppm) by 90 min on breath testing (12). It can lead to serious manifestations, such as vitamin and iron deficiency, malabsorption, and weight loss (13). IMO and ISO are detected by breath testing, through the measurement of significant increases in breath methane (CH_4_) (1, 12, 14) and hydrogen sulfide (H_2_S) (1), respectively. While IMO is associated with higher absolute abundance of *Methanobrevibacter smithii* in the small bowel (15) and stool (14) and clinical manifestations such as constipation (16) and vitamin B12 deficiency (17), and is more prevalent in IBS-C than in inflammatory bowel disease (IBD) (18), ISO is associated with the overgrowth of H_2_S producers, such as *Desulfovibrio* spp. (1, 15), and clinical symptoms, such as diarrhea (1) and abdominal pain (19).

While the identification of specific microorganisms associated with SIBO, IMO, and ISO has advanced our understanding of these GI conditions and facilitated the development of targeted treatments, no studies to date have explored differences in host small bowel gene expression within these contexts. Furthermore, it is well-established that the microbiome significantly influences host gene expression (20), and next-generation RNA sequencing offers valuable insights into the mechanisms underlying health and disease states.

Here, we aimed to (i) explore the small bowel transcriptomics profiles in SIBO, IMO, and ISO in subjects from the REIMAGINE study; and (ii) determine if mechanisms in ISO associate with findings in animals gavaged with predominant species in ISO.

## RESULTS

### Subject demographics and classification of SIBO, IMO, and ISO

The distribution of SIBO, IMO, and ISO in a group of 85 subjects identified from the REIMAGINE study is summarized in Fig. S1 and metadata with subjects’ demographics are provided in Table S1. Reasons for EGD are provided in Table S2. Small bowel transcriptomic analyses were performed for all subjects (mean age = 59 ± 14 years, female [F] = 58%, mean BMI = 26 ± 6 kg/m^2^, Caucasian [Cau] = 84%). McConkey agar culture of duodenal aspirates showed that 64 subjects had <10^3^ CFU/mL and were considered non-SIBO (mean age = 57 ± 14 years, F = 53%, BMI = 26 ± 6 kg/m^2^, Cau = 86%), while 21 had bacterial growth ≥10^3^ CFU/mL and were positive for SIBO (mean age = 65 ± 13 years, F = 71%, mean BMI = 26 ± 6 kg/m^2^, Cau = 76%). Fasting breath tests were performed for all subjects to detect CH_4_ and H_2_S levels. Twenty-two subjects were positive for IMO (CH_4_ ≥ 10 ppm) (mean age = 60 ± 14 years, F = 55%, mean BMI = 26 ± 6 kg/m^2^, Cau = 82%), and 63 subjects were classified as non-IMO (mean age = 59 ± 13 years, F = 59%, mean BMI = 26 ± 5 kg/m^2^, Cau = 84%). ISO (H_2_S ≥ 1.5 ppm) was present in 46 subjects (mean age = 60 ± 14 years, F = 52%, mean BMI = 26 ± 6 kg/m^2^, Cau = 80%), and 39 subjects were classified as non-ISO (mean age = 59 ± 14 years, F = 64%, mean BMI = 26 ± 5 kg/m^2^, Cau = 87%) (Table 1).

**TABLE 1:** Demographics^*a*^

Characteristic	McConkey agar culture (*n* = 85)			Fasted breath test (*n* = 85)								
	Non-SIBO (*n* = 64)	SIBO (*n* = 21)	*P* value	Non-IMO (*n* = 63)	IMO (*n* = 22)	*P* value	Non-ISO (*n* = 39)	ISO H_2_S ≥1.5 ppm (*n* = 46)	*P* value	NNN (*n* = 19)	ISO-only H_2_S ≥ 1.5 ppm (*n* = 28)	*P* value
Age (years)	57 ± 14	65 ± 13	0.019	60 ± 13	57 ± 15	0.466	57 ± 15	61 ± 13	0.190	56 ± 13	59 ± 13	0.408
Female	34 (53%)	15 (71%)	0.203	37 (59%)	12 (55%)	0.804	25 (64%)	24 (52%)	0.282	13 (68%)	13 (46%)	0.602
BMI (kg/m^2^)	26 ± 6	24 ± 4	0.396	26 ± 6	24 ± 4	0.453	26 ± 6	25 ± 5	0.818	27 ± 8	26 ± 5	0.957
Caucasian	55 (86%)	16 (76%)	0.401	53 (84%)	18 (82%)	0.144	34 (87%)	37 (80%)	0.734	17 (90%)	23 (82%)	0.232

^*a*^ Characteristics are presented as either mean ± standard deviation or total counts (percentage). Fisher’s exact test was performed in a 2 × 2 categorical variables comparison on SPSS v.24; otherwise, Pearson chi-square was considered. Numeric variables were compared using either a *t*-test (normal distribution) or Mann-Whitney on GraphPad Prism v.9.5.1. BMI, body mass index.

### Differentially expressed genes in ISO ± SIBO/IMO are associated with imbalances in redox, mitochondrial, and immune response pathways

**Fig 1:**
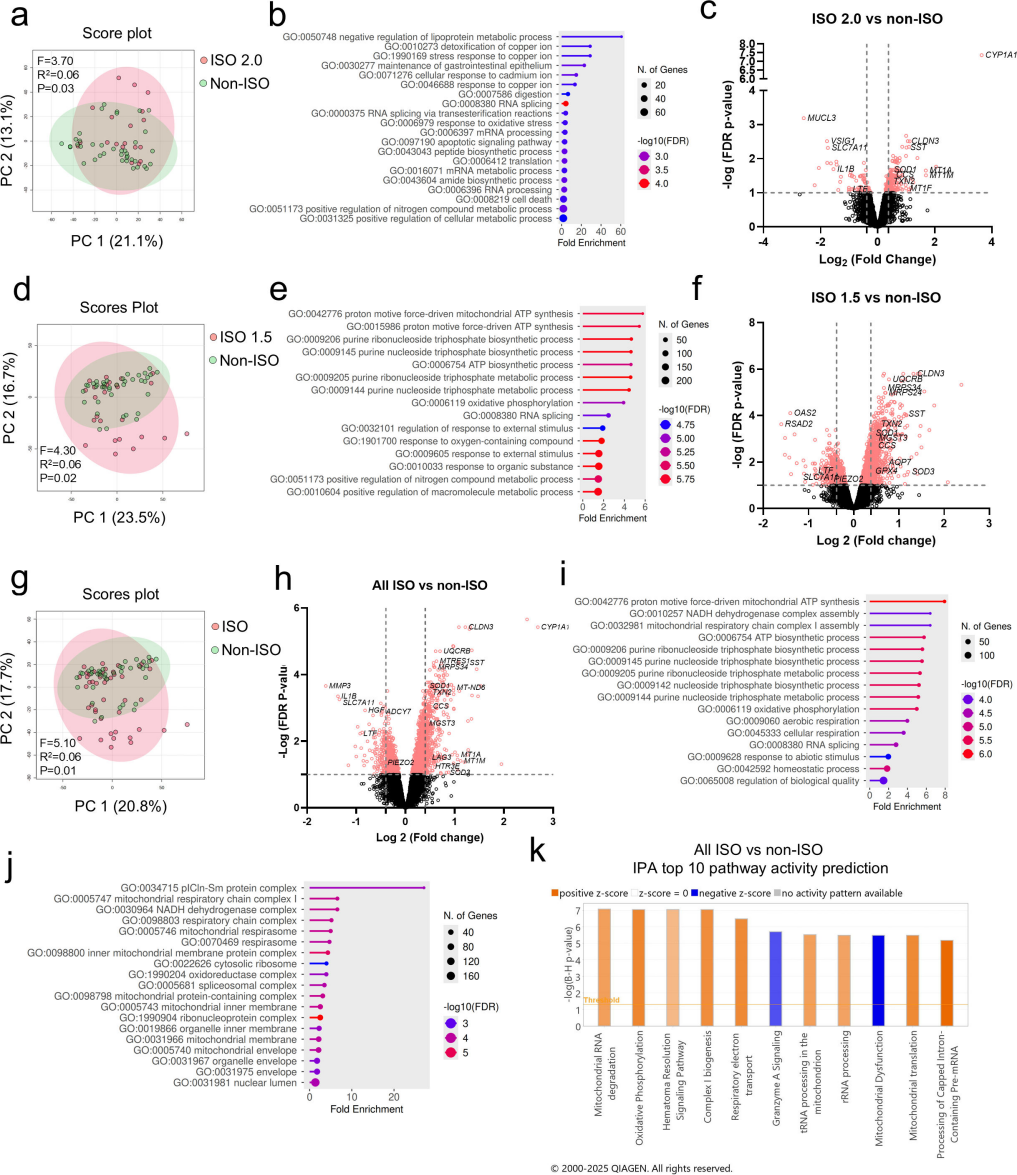
SB transcriptomics in ISO vs non-ISO. (**a**) Principal component analysis (PCA) plot showing differences in transcriptomics profiles in ISO 2.0 vs non-ISO; (**b**) top gene ontology (GO) biological processes (BPs) in ISO-2.0; (**c**) volcano plot of gene expression in ISO-2.0; (**d**) PCA plot showing differences in transcriptomics profiles in ISO 1.5 vs non-ISO; (**e**) top GO BPs in ISO-1.5; (**f**) volcano plot of gene expression in ISO-1.5; (**g**) PCA plot showing differences in transcriptomics profiles in all ISO vs non-ISO; (**h**) volcano plot of gene expression in all ISO; (**i**) top GO BPs in all ISO; (**j**) top GO cellular components (CCs) in all ISO; (**k**) activation prediction analysis of the top 10 canonical pathways in all ISO, with blue representing inhibition, and orange representing activation. DEGs are in red and have FDR < 0.1 (−log FDR > 1), and |log_2_ fold change| ≥ 0.38 (gray dotted lines were used to delimit DEGs in the volcano plots).

Using an initial threshold of H_2_S ≥ 2.0 ppm, we compared ISO 2.0 (*n* = 20) vs non-ISO (*n* = 39) subjects to explore changes in transcriptomics. To formally test whether the overall gene expression profiles were different between the groups, the permutational multivariate analysis of variance (PERMANOVA) test was applied and detected a significant difference (*F*-value = 3.70, *R*^2^ = 0.06, *P* = 0.03) (Fig. 1a). ISO-2.0 vs non-ISO had 233 differentially expressed genes (DEGs) (Table 2 and File S2), mostly associated with RNA splicing, but also with maintenance of gastrointestinal epithelium, and response to oxidative stress was one of the main pathways (Fig. 1b). Cytochrome P450 1A1 (*CYP1A1*), which can be oxidative or reductive, was the top significantly increased DEG (log_2_ fold change [Log_2_FC] = 3.6, *q* < 0.001), while mucin-like 3 (*MUCL3*) was the most significant decreased DEG (Log_2_F = −2.58, *q* < 0.001) (Table 2, Fig. 1c, and File S2). Interestingly, metallothioneins (MTs), which control redox state, were upregulated in ISO 2.0: *MT1A* (Log_2_FC = 1.68, *q* = 0.02), *MT1F* (Log_2_FC = 0.99, *q* = 0.07), *MT1G* (Log_2_FC = 0.85, *q* = 0.08), and *MT1M* (Log_2_FC = 1.69, *q* = 0.03) (Fig. 1c, Table 2, and File S2). Also, the solute carrier family 7 member 11 (*SLC7A11*), associated with control of glutathione production and ferroptosis, an iron-dependent accumulation of toxic lipid peroxides (21), was downregulated (Log_2_FC = −1.74, *q* < 0.01) (Fig. 1c, Table 2, and File S2). Due to the important effects of H_2_S on mitochondrial function and redox state (22, 23), further analyses mainly focused on genes associated with these pathways. Other relevant antioxidant DEGs were found in ISO 2.0 vs non-ISO: superoxide dismutase 1 (*SOD1*; Log_2_FC = 0.44, *q* = 0.02), copper chaperone for superoxide dismutase (*CCS*; Log_2_FC = 0.51, *q* = 0.03), and thioredoxin 2 (*TXN2*; Log_2_FC = 0.41, *q* = 0.04) (Fig. 1c, Table 2, and File S2).

**TABLE 2:** Genes of interest and key findings in ISO^*a*^

H_2_S levels (breath)	Comparisons	Key findings	Description	Log_2_FC	*q* value
≥2.0 ppm	ISO 2.0 vs non-ISO	233 DEGs	RNA splicing and response to oxidative stress	|Log_2_FC| ≥ 0.38	<0.1
		*CYP1A1* top ↑	Monooxygenase involved in xenobiotic metabolism. Can be oxidative or reductive (24, 25)	3.64	<0.001
		*MUCL3* top ↓	Mucin-like gene; mucins are involved in GI defense and inflammatory response (26, 27)	2.58	<0.001
		*SOD1*	Antioxidant enzyme (28, 29)	0.44	0.02
		*CCS*	Metalloprotein. Delivers copper to SOD1, enabling its function (12)	0.51	0.03
		*TXN2*	Regulates mitochondrial redox and ROS production (30)	0.41	0.04
		*SLC7A11*	Supports GSH generation via c ystine cellular import (31); controls ferroptosis (21)	−1.74	<0.01
		*MT1A*	Detoxifies metal ions; protects cells from oxidative stress and inflammation (32)	1.68	0.02
		*MT1M*	Detoxifies metal ions; protects cells from oxidative stress and inflammation (32)	1.69	0.03
		*MT1G*	Detoxifies metal ions; protects cells from oxidative stress and inflammation (32)	0.85	0.08
		*MT1F*	Detoxifies metal ions; protects cells from oxidative stress and inflammation (32)	0.99	0.07
		*SST*	Somatostatin. Inhibits secretory activity and GI motility, and modulates pain (33)	1.00	<0.01
		*LTF*	Antimicrobial peptide (34)	−1.00	0.09
		*IL1B*	Pro-inflammatory cytokine (35)	−1.54	0.02
	ISO only 2.0 vs NNN	17 DEGs	Response to stress, and immune response	|Log_2_FC| ≥ 0.38	<0.1
		*CYP1A1* top ↑	Monooxygenase involved in xenobiotic metabolism. Can be oxidative or reductive (24, 25)	5.06	<0.001
		*SLC7A11* top ↓	Supports GSH generation via cystine cellular import (36); controls ferroptosis (21)	−2.46	<0.01
		*IL1B*	Pro-inflammatory cytokine (35)	−2.14	0.03
1.5 ppm ≤ H_2_S > 2.0 ppm	ISO 1.5 vs non-ISO	923 DEGs	Response to oxygen-containing compound; mitochondrial ATP synthesis	|Log_2_FC| ≥ 0.38	<0.1
		*SOD1*	Antioxidant enzyme (28, 29)	0.40	<0.001
		*SOD3*	Secretory antioxidant enzyme (37)	1.20	0.02
		*TXN2*	Regulates mitochondrial redox and ROS production (30)	0.52	<0.001
		*CCS*	Metalloprotein. Delivers copper to SOD1, enabling its function (12)	0.45	<0.01
		*GPX4*	Belongs to the glutathione peroxidase family. Protects cells from oxidative damage (38)	0.39	0.02
		*IL1B*	Pro-inflammatory cytokine (35)	−1.26	0.01
		*MGST3*	Enzyme with glutathione-dependent peroxidase activity (39)	0.5	<0.001
		*SLC7A11*	Supports GSH generation via cystine cellular import (36); controls ferroptosis (21)	−1.10	0.03
		*LTF*	Antimicrobial peptide; sequestrates iron (34)	−0.86	0.03
		*FTH1*	Ferritin heavy chain; involved with iron storage; protects from ferroptosis (40)	0.39	0.03
		*AQP7*	Aquaporin 7; regulates fluid homeostasis (41)	0.69	0.01
		*PIEZO2*	Affects gut motility; knockout mice show increased stool water content and faster transit time (42)	−0.50	0.05
	ISO only 1.5 vs NNN	270 DEGs	Activation of mitochondrial respiration and inhibition of immune response	|Log_2_FC| ≥ 0.38	<0.1
		*SLC7A11*	Supports GSH generation via cystine cellular import (36); controls ferroptosis (21)	−2.13	<0.01
		*SST*	Somatostatin. Inhibits secretory activity and GI motility, and modulates pain (33)	1.38	<0.01
		*AQP7*	Aquaporin 7; regulates fluid homeostasis (41)	0.97	0.01
		*IL1B*	Pro-inflammatory cytokine (35)	−2.08	<0.01
		*CTH*	Endogenous H_2_S-producing enzyme (43)	−0.57	0.06
H_2_S ≥ 1.5 ppm	All ISO (1.5 + 2.0) vs non-ISO	643 DEGs	Mitochondrial ATP synthesis; cellular respiration	|Log_2_FC| ≥ 0.38	<0.1
		*CYP1A1* top ↑	Monooxygenase involved in xenobiotic metabolism. Can be oxidative or reductive (24, 25)	2.68	<0.001
		*MMP3* top ↓	Matrix metalloproteinase involved in wound healing and inflammation (44)	−1.62	<0.001
		*SOD1*	Antioxidant enzyme (28, 29)	0.42	<0.001
		*CCS*	Metalloprotein. Delivers copper to SOD1, enabling its function (12)	0.48	<0.001
		*TXN2*	Regulates mitochondrial redox and ROS production (30)	0.47	<0.001
		*MT1A*	Detoxifies metal ions; protects cells from oxidative stress and inflammation (32)	1.05	0.03
		*MT1M*	Detoxifies metal ions; protects cells from oxidative stress and inflammation (32)	1.12	0.03
		*SLC7A11*	Supports GSH generation via cystine cellular import (31); controls ferroptosis (21)	−1.34	<0.001
		*PIEZO2*	Affects gut motility; knockout mice show increased stool water content and faster transit time (45)	−0.43	0.04
		*IL1B*	Pro-inflammatory cytokine (35)	−1.37	<0.001
		*LTF*	Antimicrobial peptide (34)	−0.92	<0.01
		*FTH1*	Ferritin heavy chain; involved with iron storage; protects from ferroptosis (40)	0.41	<0.01
		*HTR3E*	Serotonin receptor involved in IBS-D phenotype (46)	0.53	0.07
		*LAG3*	Marker of T-cell exhaustion; involved in ulcerative colitis (47, 48)	0.47	0.03
	ISO only (1.5 + 2.0) vs NNN	132 DEGs	Decreased expression of immune response genes; increased expression of genes associated with mitochondrial ATP production	|Log_2_FC| ≥ 0.38	<0.1
		*CYP1A1* top ↑	Monooxygenase involved in xenobiotic metabolism. Can be oxidative or reductive (10, 11)	3.97	<0.001
		*SLC7A11 top* ↓	Supports GSH generation via cystine cellular import (36); controls ferroptosis (40)	−2.25	<0.001
		*IL1B*	Pro-inflammatory cytokine (35)	−2.10	<0.001
		*CTH*	Endogenous H_2_S-producing enzyme (43)	−0.58	0.03
		*SST*	Somatostatin. Inhibits secretory activity and GI motility, and modulates pain (33)	1.13	<0.01

^*a*^ DEGs, differentially expressed genes; Log_2_FC, log_2_ fold change; *AQP7*, aquaporin 7; *CCS*, copper chaperone for superoxide dismutase; *CTH*, cystathionine gamma-lyase; *CYP1A1*, cytochrome P450 1A1; *FTH1*, ferritin heavy polypeptide 1; *GPX4*, glutathione peroxidase 4; *HTR3E*, 5-hydroxytryptamine receptor 3E; *IL1B*, interleukin-1 beta; *LAG3*, lymphocyte activation gene 3; *LTF*, lactoferrin; *MMP3*, matrix metalloproteinase 3; *MGST3*, microsomal glutathione S-transferase 3; *MMP3*, matrix metalloproteinase 3; *MT1A* (-B, -F, -G, -M), metallothionein 1A-M; *MUCL3*, mucin-like 3; *PIEZO2*, piezo type mechanosensitive ion channel component 2; *SLC7A11*, solute carrier family 7 member 11; *SOD1-3*, superoxide dismutase 1-3; *SST*, somatostatin; *TXN2*, thioredoxin 2; *UTG1A6*, UDP glucuronosyltransferase family 1 member A6.

We then included subjects with H_2_S between 1.5 and 1.9 ppm (ISO 1.5) in the analysis to explore if levels below 2.0 ppm exhibited alterations in relevant pathways. There was a significant difference in the overall transcriptomics profiles between ISO 1.5 (*n* = 26) vs non-ISO (*n* = 39) (*F*-value = 4.3, *R*^2^ = 0.06, *P* = 0.02) (Fig. 1d). A total of 923 DEGs were identified (File S2), and response to oxygen-containing compound was one of the most significant associated biological processes (Fig. 1e). *SOD1* (Log_2_FC = 0.40, *q* < 0.001), *TXN2* (Log_2_FC = 0.52, *q* < 0.001), *CCS* (Log_2_FC = 0.45, *q* < 0.01), and *SLC7A11* (Log_2_FC = −1.10, *q* = 0.03) were similarly affected, and glutathione peroxidase 4 (*GPX4*, Log_2_FC = 0.39, *q* = 0.02), *SOD3* (Log_2_FC = 1.20, *q* = 0.02), and microsomal glutathione S-transferase 3 gene (*MGST3*, Log_2_FC = 0.47, *q* < 0.001) were also dysregulated, suggesting altered redox state can occur below the 2.0 ppm level (Fig. 1f, Table 2, and File S2).

ISO 1.5 and 2.0 ppm were then combined (*n* = 46) and compared to non-ISO subjects (*n* = 39). A significant difference in transcriptomics profiles between the groups was detected (*F* = 5.1, *R*^2^ = 0.06, *P* = 0.008) (Fig. 1g), and 643 DEGs were identified (File S2). *CYP1A1* was the most significantly upregulated gene (Log_2_FC = 2.68, *q* < 0.001), while *MMP3* was the least significantly expressed (Log_2_FC = −1.62, *q* < 0.001), alongside *IL1B* (Log_2_FC = −1.37, *q* < 0.001) and *SLC7A11* (Log_2_FC = −1.34, *q* < 0.001) (Fig. 1h, Table 2, and File S2). Antioxidant DEGs were *SOD1* (Log_2_FC = 0.42, *q* < 0.001), *CCS* (Log_2_FC = 0.48, *q* < 0.001), *MT1A* (Log_2_FC = 1.05, *q* = 0.03), *MT1M* (Log_2_FC = 1.12, *q* = 0.03), and *TXN2* (Log_2_FC = 0.47, *q* < 0.001) (Fig. 1h, Table 2, and File S2). Top biological processes in all ISO vs non-ISO included mitochondrial ATP synthesis as the most significant pathway, alongside response to abiotic stimulus, and RNA splicing (Fig. 1i). Overall, the DEGs in ISO vs non-ISO were associated with the ribonucleoprotein complex (q < 0.0001) and inner mitochondrial compartments (Fig. 1j). Ingenuity pathway analysis (IPA) predicted activation of mitochondrial RNA degradation, oxidative phosphorylation, and complex I biosynthesis (B-H *P* value < 0.05) (Fig. 1k).

### ISO-only DEGs are associated with imbalances in mitochondrial respiration, immune response, and endogenous H_2_S production

**Fig 2:**
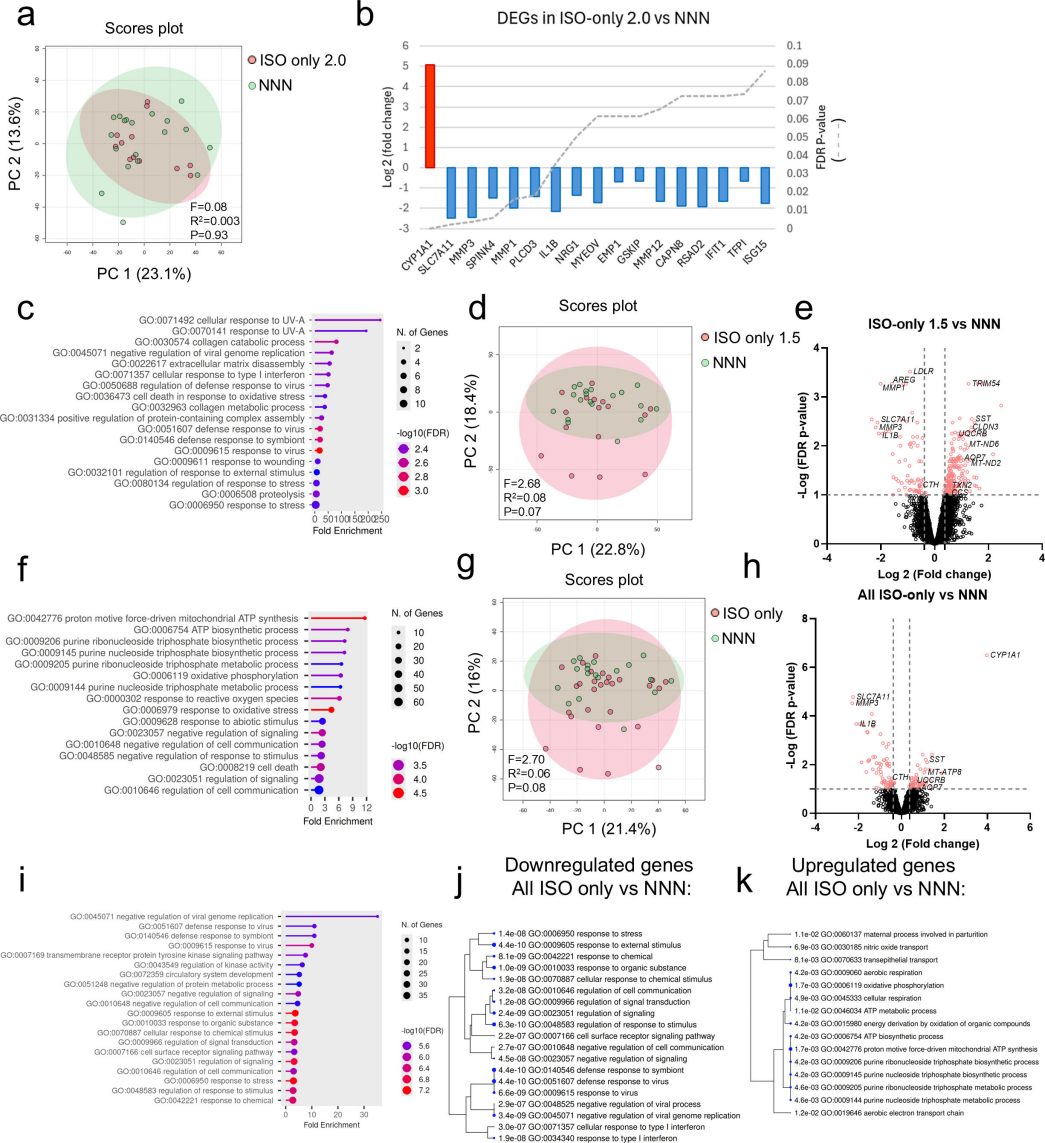
SB transcriptomics in ISO-only vs non-SIBO, non-IMO, non-ISO (NNN). (**a**) PCA plot showing transcriptomics profiles distribution in ISO-only 2.0 vs NNN; (**b**) DEGs in ISO-only 2.0; **(c**) top GO BPs in ISO-only 2.0; (**d**) PCA plot showing transcriptomics profiles distribution in ISO-only 1.5 vs NNN; (**e**) volcano plot of the gene expression in ISO-only 1.5 vs NNN; (**f**) top GO BPs in ISO-only 1.5; (**g**) PCA plot showing transcriptomics profiles in all ISO only vs NNN; (**h**) volcano plot of the gene expression in all ISO-only; (**i**) top GO BPs in ISO-only 1.5; (**j**) top GO BPs associated with downregulated genes in all ISO-only; (**k**) top GO BPs associated with upregulated genes all ISO-only. DEGs are in red and have FDR < 0.1 (−log FDR > 1), and |log_2_ fold change| ≥ 0.38 (gray dotted lines were used to delimit DEGs in the volcano plots).

In addition to ISO vs non-ISO comparisons, we explored changes in transcriptomics when ISO was not accompanied by SIBO and/or IMO. Subjects with ISO only were compared to subjects without ISO, IMO, and SIBO (NNN). No differences in demographics were identified in ISO-only vs NNN comparisons (Table 1 and Table S1). Despite no overall changes in transcriptomics profiles in ISO only 2.0 (*n* = 12) vs NNN (*n* = 19) (*F*-value = 0.08, *R*^2^ = 0.003, *P* value = 0.93) (Fig. 2a), 17 DEGs were identified, and *CYP1A1* remained the top significantly increased DEG (Log_2_FC = 5.06, *q* < 0.001), and *SLC7A11* was the top decreased DEG (FC = −2.46, *q* < 0.01) (Fig. 2b, Table 2, and File S2). Most DEGs were associated with response to stress, and several biological processes involved response to virus (Fig. 2c). ISO only 1.5 (*n* = 16) vs NNN overall transcriptomics profiles showed a tendency to be significantly different (*F*-value = 2.68, *R*^2^ = 0.08, *P* value = 0.07) (Fig. 2d), and 270 DEGs were identified (File S2); as in ISO, *SLC7A11* was decreased (Log_2_FC = −2.13, *q* < 0.01), along with the dysregulation of several DEGs identified in ISO 1.5 comparisons (Fig. 2e, Table 2, and File S2). Moreover, there was a decrease (Log_2_FC = −0.57, *q* = 0.06) in cystathionine gamma-lyase (*CTH*, an endogenous H_2_S-producing enzyme) in ISO only 1.5 vs NNN (Fig. 2e and Table 2 and File S2), showing that subjects with exhaled ISO-only below 2.0 ppm already had important changes in transcriptomics. Aquaporin 7, which controls the transport of water and small molecules through the intestinal epithelium, was upregulated (*AQP7;* Log_2_FC = 0.97, *q* = 0.01) (Table 2, Fig. 2f, and File S2). Mitochondrial ATP synthesis and response to oxidative stress/oxygen species persisted as top biological processes in ISO only 1.5 (Fig. 2f).

When all ISO-only subjects (1.5 + 2.0, *n* = 28) were compared to NNN, despite no overall difference in transcriptomics profiles being found between the groups (*F*-value = 2.7, *R*^2^ = 0.06, *P* = 0.08) (Fig. 2g), 132 DEGs were identified (Fig. 2h and File S2). *SLC7A11* (Log_2_FC = −2.25, *q* < 0.001) was the most significant downregulated gene, and *CYP1A1* was the most expressed gene (Log_2_FC = 3.97, *q* < 0.001) (Fig. 2h, Table 2, and File S2). *CTH* was confirmed as a downregulated gene in all ISO only (Log_2_FC = −0.58, *q* = 0.03), along with *IL1B* (Log_2_FC = −2.10, *q* < 0.001). *AQP7* was still an upregulated gene (Log_2_FC = 0.76, *q* = 0.08) (Fig. 2h, Table 2, and File S2). DEGs in all ISO only subjects were associated with stress responses, response to virus (Fig. 2i), and immune pathways that were especially controlled by the downregulated genes (Fig. 2j), while upregulated genes were associated with mitochondrial ATP synthesis and oxidative phosphorylation (Fig. 2k). When FDR < 0.05 was applied to the ISO vs non-ISO and ISO-only vs NNN comparisons, 572 and 67 DEGs were identified, respectively (File S2); the biological processes associated with these DEGs are described in Fig. S2.

### Exploratory transcriptomics in IMO-only identifies DEG regulators of lipid metabolism, cell cycle, and antimicrobial response

**Fig 3:**
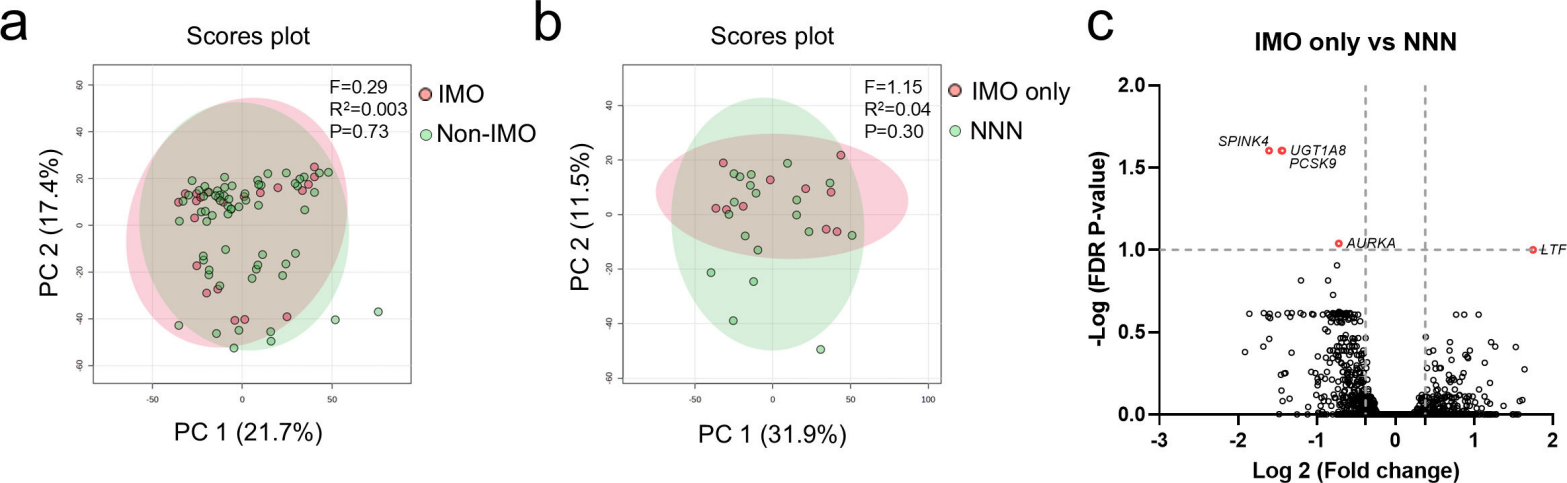
SB transcriptomics in IMO. (**a**) PCA plot showing the overall transcriptomics profiles distribution in IMO vs non-IMO; (**b**) PCA plot showing the overall transcriptomics profiles distribution in IMO only vs NNN; (**c**) volcano plot of the gene expression in IMO only vs NNN. DEGs are in red and have FDR < 0.1 (−log FDR > 1), and |log_2_ fold change| ≥ 0.38 (gray dotted lines were used to delimit DEGs in the volcano plots).

Comparing IMO (*n* = 22) vs non-IMO (*n* = 63), there was no change in demographics (Table S1) and no overall differences in transcriptomics profiles (*F*-value = 0.29, *R*^2^ = 0.003, *P* = 0.73) (Fig. 3a). Also, no genes were differentially expressed (File S2). When subjects with IMO only (*n* = 10) were compared to NNN, no differences in demographics (Table S1) and overall transcriptomics profiles were observed (*F*-value = 1.15, *R*^2^ = 0.04, *P* = 0.30) (Fig. 3b), but five DEGs were identified: the proprotein convertase subtilisin/kexin type 9 (*PCSK9*, Log_2_FC = −1.44, *q* = 0.02), associated with small bowel cholesterol absorption; the UDP-glucuronosyltransferase family 1 member A8 (*UGT1A8*, Log_2_FC = −1.43, *q* = 0.02), which catalyzes glucuronidation of steroid hormones, bilirubin, and drugs; the serine peptidase inhibitor, Kazal type 4 (*SPINK4*, Log_2_FC = −1.60, *q* = 0.02), associated with gut protection against proteases, such as trypsin; the aurora kinase A (*AURKA*, Log_2_FC = −0.72, *q* = 0.09), which controls cell cycle and proliferation; and lactoferrin (*LTF*, Log_2_FC = 1.74, *q* = 0.09), which is bacteriostatic and upregulated when microbial growth needs to be controlled (Fig. 3c and File S2).

### Exploratory SIBO transcriptomics reveals dysregulation of genes associated with cell cycle progression and innate immune response, which are dependent on age

**Fig 4:**
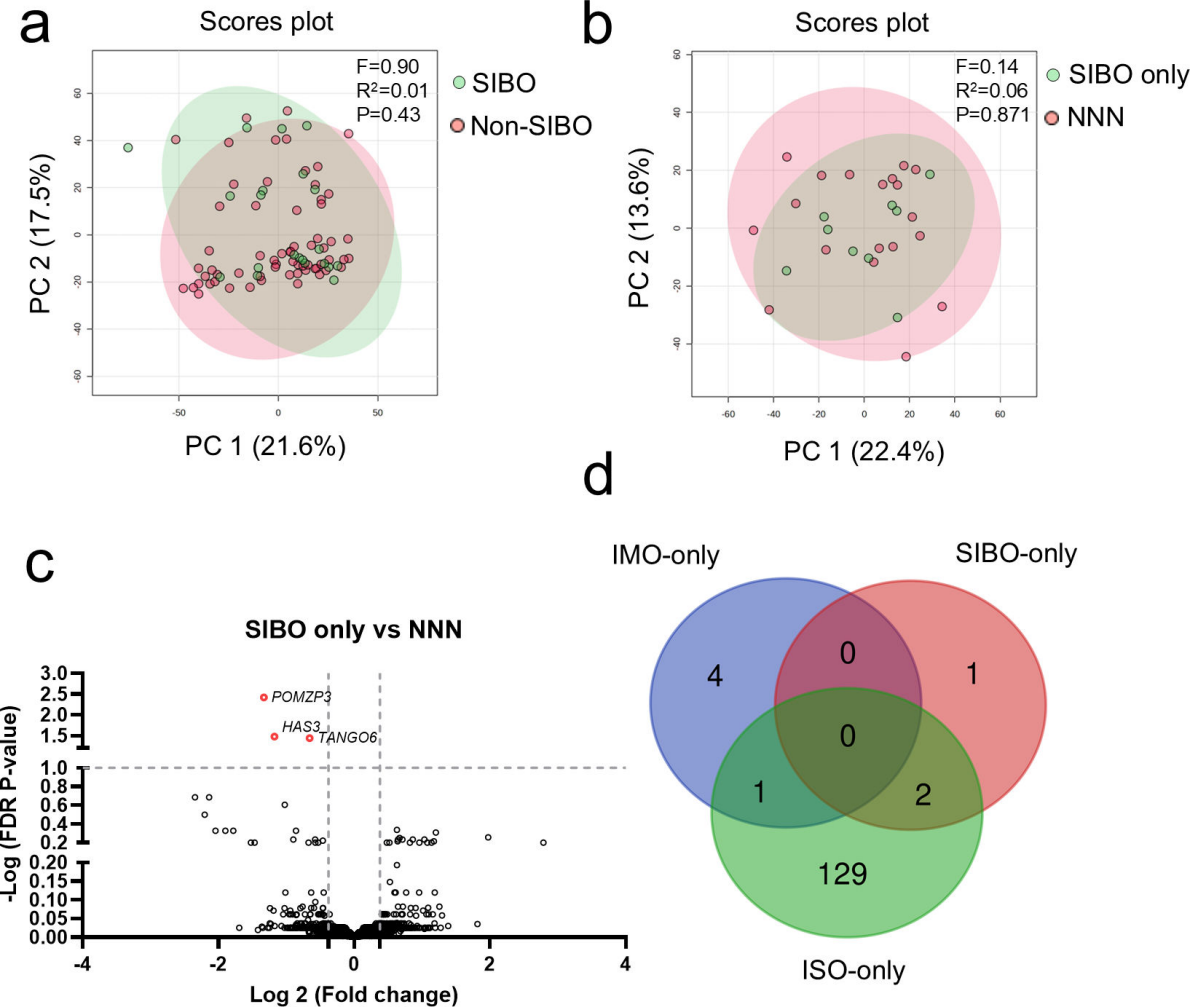
SB transcriptomics in SIBO vs non-SIBO and SIBO-only vs NNN, and common DEGs among ISO-only, IMO-only, and SIBO-only. (**a**) PCA plot showing the overall transcriptomics profiles distribution in SIBO vs non-SIBO; (**b**) PCA plot showing the overall transcriptomics profiles distribution in SIBO only vs NNN; (**c**) volcano plot of the gene expression in SIBO only vs NNN; (**d**) Venn diagram of the DEGs in IMO only, SIBO only, and ISO only. DEGs are in red and have FDR < 0.1 (−log FDR > 1), and |log_2_ fold change| ≥ 0.38 (gray dotted lines were used to delimit DEGs in the volcano plots).

SIBO (*n* = 21) vs non-SIBO (*n* = 64) showed no changes in overall transcriptomics profiles (*F*-value = 0.90, *R*^2^ = 0.01, *P* = 0.43) (Fig. 4a), but there was a downregulation in the gene expression of the solute carrier family 30 member 2 (*SLC30A2*, Log_2_FC = 2.02, *q* = 0.06), which controls the levels of zinc in intracellular granules, especially in Paneth cells, and improves the secretion of antimicrobial peptides, such as defensins and lysozyme (49) (File S2). When adjusted by age, none of the genes were differentially expressed in SIBO vs non-SIBO (*q* > 0.1, data not shown). In SIBO only (*n* = 9) vs NNN (*n* = 19), no changes in demographics (Table S1) and overall transcriptomics profiles were observed (*F*-value = 0.14, *R*^2^ = 0.06, *P* = 0.871) (Fig. 4b), but three DEGs were identified (independent of age): the *POM121* and *ZP3* fusion (*POMZP3*, Log_2_FC = −1.33, *q* < 0.01), with no clearly defined function; the transport and Golgi organization protein 6 (*TANGO6*, Log_2_FC = −0.65, *q* = 0.03), which regulates cell cycle and proliferation (50); and hyaluronan synthase 3 (*HAS3*, Log_2_FC = −1.17, *q* = 0.03), which produces hyaluronan, a major component of the extracellular matrix (51) (Fig. 4c). SIBO-only, IMO-only, and ISO-only comparisons did not share a single common DEG (Fig. 4d). The average percentage of mapped reads to the human reference genome hg38 was 90.1% for all human samples (File S3). Moreover, no batch effect was observed across human sequencing runs (Fig. S3). A Venn diagram comparing all groups is shown in Fig. S4.

### H_2_S-producer-gavaged rats exhibit small bowel transcriptomic effects consistent with findings in ISO

**Fig 5:**
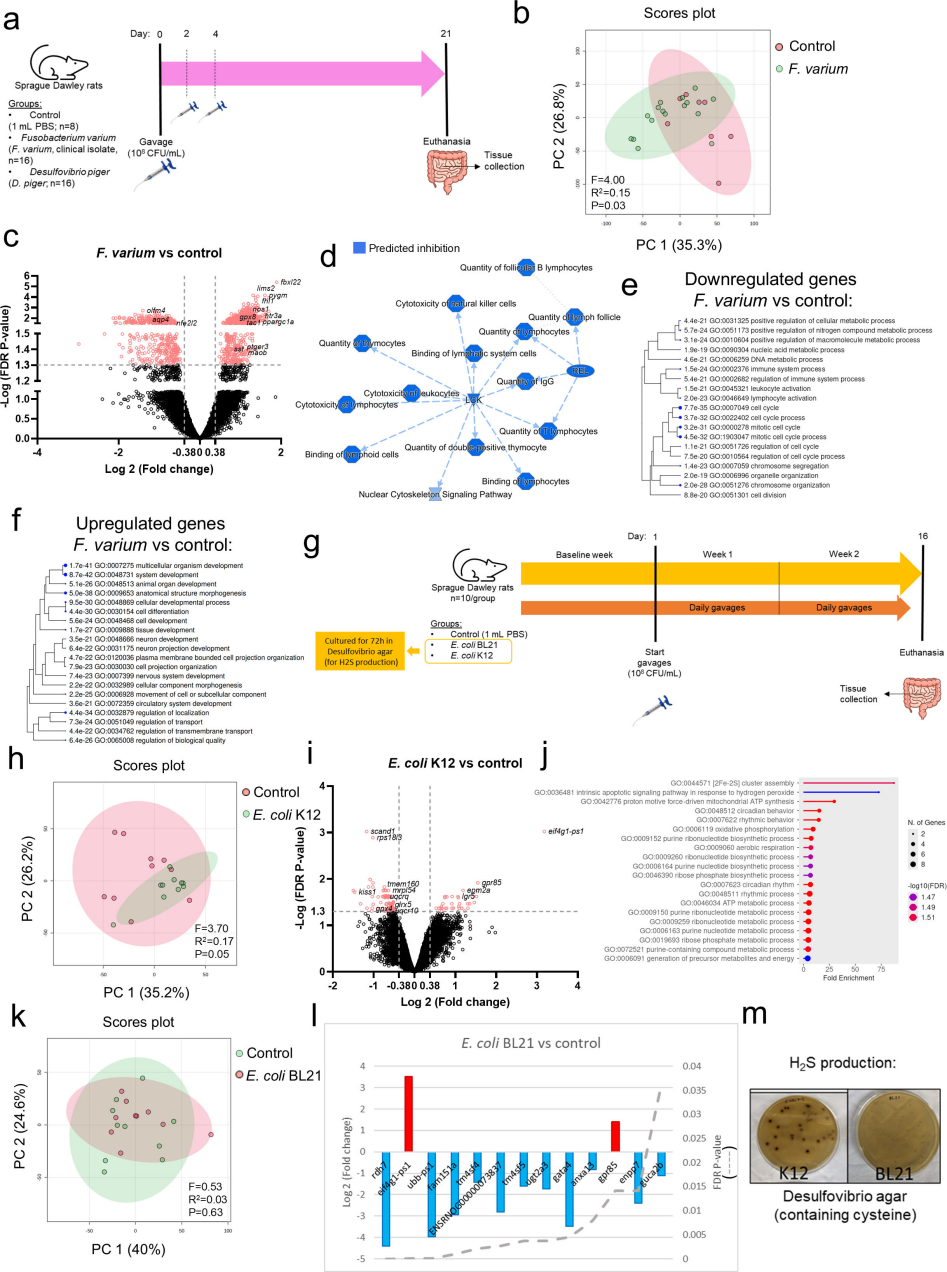
SB transcriptomics in H_2_S-producer-gavaged rats. (**a**) Model for *D. piger* and *F. varium* gavages in rats; (**b**) PCA plot showing differences in transcriptomics profiles in *F. varium*-gavaged rats vs controls; (**c**) volcano plot of the gene expression in *F. varium* vs controls; (**d**) IPA core analysis summary of findings associated with the DEGs in *F. varium* vs controls. Blue means prediction of inhibition; (**e**) top GO BPs associated with downregulated genes in *F. varium* group; (**f**) top GO BPs associated with upregulated genes in *F. varium* group; (**g**) model for *E. coli* K12 and *E. coli* BL21 gavages in rats; (**h**) PCA plot showing differences in transcriptomics profiles in *E. coli* K12-gavaged rats vs controls; (**i**) volcano plot of the gene expression in *E. coli* K12 vs controls; (**j**) top GO BPs in *E. coli* K12*-*gavaged rats; (**k**) PCA plot showing differences in transcriptomics profiles in *E. coli* BL21-gavaged rats vs controls; (**l**) DEGs in *E. coli* BL21-gavaged rats; (**m**) *E. coli* K12 and BL21 bacterial cultures on *Desulfovibrio* agar for H_2_S production investigation. DEGs are in red and have FDR < 0.05 (−log FDR > 1.3), and |Log_2_ fold change| ≥ 0.38 (gray dotted lines were used to delimit DEGs in the volcano plots).

Rats gavaged with known H_2_S producers, *Fusobacterium varium* (clinical isolate) and *Desulfovibrio piger*, and rats gavaged with two *Escherichia coli* strains as controls, were used to explore their effects on rat small bowel transcriptomics. First, rats were gavaged every other day for 3 days with 1 mL of PBS (control, *n* = 8) or 10^8^ CFU/mL of *F. varium* (*n* = 16) or *D. piger* (*n* = 16), as described in Villanueva-Millan et al. (2) (Fig. 5a). *F. varium*-gavaged animals showed significant differences in overall transcriptomics profile vs controls (*F*-value = 4.0, *R*^2^ = 0.15, *P* = 0.03) (Fig. 5b) and had 1,244 DEGs (Fig. 5c), including downregulation of the strictly controlled nuclear factor E2-related factor 2 (*nfe2l2*; Log_2_FC = −0.62, *q* = 0.03), which regulates the expression of several antioxidant genes, such as the upregulated *gpx8* (52) (Log_2_FC = 0.87, *q* = 0.03). Other findings of interest included the dysregulation of aquaporin 4 (*aqp4*, Log_2_FC = −1.26, *q* = 0.01), somatostatin (*sst*; FC = 0.84, *q* = 0.04), prostaglandin receptor 3 (*ptger3*; Log_2_FC = 0.87, *q* = 0.03), monoamine oxidase b (*maob*, Log_2_FC = 0.81, *q* = 0.03), and the serotonin receptor *htr3a* (Log_2_FC = 1.50, *q* = 0.01), suggesting dysregulation in water homeostasis (53), gut motility (33), altered redox balance (54), and IBS-associated mechanisms (46, 55) (Fig. 5c and File S4). Moreover, the peroxisome proliferator-activated receptor gamma coactivator 1-alpha (*ppargc1a*) was upregulated (Log_2_FC = 0.85, *q* = 0.03), suggesting increased mitochondrial mass and antioxidant defense control (56) (Fig. 5c and File S4). The summary of findings with all DEGs in the core analysis on IPA in *F. varium* vs control was an overall dysregulation of the immune response, with a broad downregulation of genes associated with those immune pathways (Fig. 5d and 5e), while upregulated genes were associated with cell/organ differentiation and development (Fig. 5f). Comparing *D. piger*-gavaged animals (*n* = 15) to controls (*n* = 8) (Fig. 5d), no DEGs were identified (File S4). Lastly, rats were gavaged daily for 15 days with 10^8^ CFU/mL of *E. coli* BL21 (*n* = 10), or *E. coli* K12, a facultative H_2_S producer (*n* = 10), and were compared to controls (1 mL of PBS, *n* = 10) (Fig. 5g). Overall differences in the transcriptomics profiles of *E. coli* K12-gavaged rats vs controls were close to significance (Fig. 5h), and 137 DEGs were identified (Fig. 5i and File S4), mostly downregulated and associated with, among others, oxidative phosphorylation, ATP synthesis, iron regulation, and response to hydrogen peroxide (Fig. 5j). In contrast, rats gavaged with *E. coli* BL21 (*n* = 10), which shares >99% sequence identity over approximately 92% of the *E. coli* K12 genome (57), did not show overall differences in transcriptomics profile compared to the control group (Fig. 5k), and had 13 DEGs (Fig. 5l and File S4), which were not associated with the main mechanisms in ISO and/or H_2_S-producers gavages in rats, such as translation, splicing, broad immune downregulation, mitochondrial function, or redox balance. Furthermore, most DEGs were unique to each *E. coli* group (Fig. S5). H_2_S production by both *E. coli* strains was analyzed by culture on *Desulfovibrio* agar, and only *E. coli* K12 was H_2_S+ (Fig. 5m), suggesting that H_2_S production may be involved in the differences in gene expression. Moreover, these results suggest that daily gavages with H_2_S-producing ATCC strains may be preferred for the examination of differences in gene expression, and that *D. piger* effects on gene expression are more pronounced in the large intestine. No batch effect in RNA sequencing was identified in *F. varium* or *D. piger* vs the control cohort (Fig. S6), and the average percentage of reads mapped to the rat reference genome GRCr8 was >83% for *F. varium, D. piger*, and the *E. coli* cohorts (File S3).

## DISCUSSION

In this exploratory study, we describe changes in small bowel transcriptomics in subjects with ISO, IMO, and SIBO, and suggest that findings in ISO are associated, at least in part, with bacterial H_2_S effects. ISO subjects and H_2_S producer-gavaged rats exhibited alterations in the expression of genes related to H_2_S-controlled mechanisms, such as mitochondrial function and redox balance, accompanied by changes in overall cellular transcription, splicing, immune response, liquid homeostasis, hypersensitivity, and transit time; these mechanisms were not associated with DEGs in rats gavaged with *E. coli* BL21, a non-H_2_S producer. Moreover, SIBO-only, IMO-only, and ISO-only comparisons did not share a single common DEG.

H_2_S is a gasotransmitter and an endogenous signaling molecule that easily diffuses into cells; for this reason, its levels are tightly regulated in the body (23). High or low H_2_S levels are harmful, and H_2_S levels are controlled by mitochondrial oxidation and the promotion of ATP synthesis, which are inhibited by its toxicity (23). Here, we found that the transcriptional profile of the small bowel of ISO subjects with exhaled H_2_S levels ≥ 1.5 ppm, and the transcriptional profile of the small bowel of rats briefly exposed to *F. varium* clinical isolate, already show changes in the expression levels of mitochondrial electron transport chain complexes (*cox6a2*, *ndufa4l2*, *atp5me*, *atp5mpl*) or mitochondrial biogenesis upstream regulators, such as *ppagc1a* in rats, suggesting an upregulation in mitochondrial respiration and mass. While this may reflect increased energy demand or compensation, mitochondrial respiration also produces reactive oxygen species (ROS). Moreover, altered mitochondrial function associated with dysregulation in antioxidative response, especially by downregulation of *SLC7A11*, combined with upregulation of *CYP1A1*, a major ROS producer in the gut (36), suggests compromised antioxidant defense and increased cell vulnerability to lipid peroxidation, and potential ferroptosis in ISO, since SLC7A11 transports cystine into the cells and promotes glutathione synthesis (21). Interestingly, *FTH1*, the ferritin heavy chain 1 that inhibits ferroptosis (40), was mildly upregulated in ISO subjects, suggesting a potential counterbalance.

H_2_S can be produced endogenously by enzymes, such as cystathionine β-synthase (*CBS*), cystathionine γ-lyase (*CSE*, or *CTH*), and 3-mercaptopyruvate sulfurtransferase (*3-MST*) (23); however, H_2_S levels in the body can be highly impacted by the gut microbiome, more specifically by H_2_S-producing bacteria, such as *F. varium* (58, 59). Interestingly, unlike ISO subjects with or without SIBO/IMO, ISO-only subjects did not exhibit dysregulation in *GPX4*, *SOD1*, *TNX2,* and other antioxidant genes; they exhibited reductions in *CTH* that, combined with the decrease in *SLC7A11* and upregulation of *CYP1A1*, suggest compromised redox balance without compensation. Furthermore, rats gavaged with *F. varium* exhibited reduced *nfe2l2,* an important regulator of antioxidative response (60), which is strictly controlled. This downregulation of *SLC7A11* in ISO, and *nfe2l2* in H_2_S-producer-gavaged animals, suggests reduced glutathione antioxidation, which has been linked to H_2_S toxicity (61).

Other mechanisms that were broadly affected in ISO included splicing, translation/transcription, and immune response. H_2_S can directly affect the expression of splicing factors, such as *HNRNPD* and *SRSF2*, and inhibit cell senescence (62, 63); moreover, H_2_S affects splicing, transcription, and other mechanisms through protein persulfidation. Here, we found that DEGs associated with splicing in ISO involved upregulation of several genes, especially the small nuclear ribonucleoproteins (*SNRPs*) (64), and translation/transcription were affected by upregulation of *SNRPs (SNRP1*, *SNRPD3*, *SNRPF*), but also ribosomal proteins (*RPLs* and *RPSs*, *or MRPSs*) (65); it is possible that persulfidation, as an indirect mechanism, is modulating the expression of these genes.

Immune response genes, especially those involved with pro-inflammatory responses, were ubiquitously inhibited in ISO; *IL1B* was downregulated in all ISO comparisons, along with *TLR4-6* and *PTGES2*, especially in ISO 1.5, suggesting reduced expression of pro-inflammatory effectors by M1 macrophages (45). Interestingly, increased *IL1B* expression in rectal biopsies was previously associated with IBS, particularly post-infectious IBS (66). Moreover, increased circulating *IL1B* was found in women with IBS and extra-intestinal co-morbidity (67). However, chronic exposure to microbial disruptors, such as H_2_S producers, may contribute to T-cell exhaustion, since H_2_S controls Treg differentiation (68). This is supported by the upregulation of *LAG3*, a marker of T-cell exhaustion (69), in ISO vs non-ISO. On the other hand, the overall downregulation of genes associated with immune response in ISO, and also in *F. varium* rats, may be a result of rapid mRNA vs protein turnover, or bulk RNAseq dilution of findings in immune cells.

We previously showed that ISO is associated with IBS-D (1), and that rats gavaged with *F. varium* and *D. piger* have increased stool water content, a diarrhea-like phenotype (2). Here, *F. varium-*gavaged rats’ transcriptomics was associated with neural signaling gene dysregulation, such as the upregulation of *gja1*, *cacna1b*, *gnao1*, and *chrm2*, which are linked to cAMP, water flux control, and motility stability (70). Not surprisingly, increased stool water content was previously found at all time points in *F. varium*-gavaged rats (2). Moreover, these rats also exhibited alterations in other genes associated with gut motility, such as nitric oxide synthase 1 (*nos1*), and *sst* (33, 71), visceral hypersensitivity, such as tachykinin 1 (*tac1,* a precursor of substance P) (72), and IBS mechanisms, such as *htr3a* (46, 55). *SST* was also upregulated in ISO 1.5 and all ISO vs non-ISO, and increased *EDN1*, *MDK*, and *AQP7*, all linked to fluid homeostasis (41, 73, 74), suggesting a link between these genes and diarrhea. *HTR3E,* another serotonin receptor associated with IBS-D phenotypes, was also upregulated in ISO vs non-ISO (46). In addition, dysregulation of colonic aquaporins, such as aquaporin 7, has been associated with diarrhea in inflammatory bowel disease (75, 76). *PIEZO2* downregulation in ISO may also contribute to loosened stools and faster transit time in subjects with higher exhaled H_2_S, as *PIEZO2*-mutated mice present a diarrhea-like phenotype, as previously reported (42), and colonic *PIEZO2* gene expression has been shown to be modulated by *F. varium* in IBS-D subjects (77).

While ISO exhibits wide changes in transcriptomics, potentially due to the broad effects of H_2_S on gene expression (23), transcriptomic changes in IMO and SIBO were less pronounced or nonexistent. IMO-only was associated with impaired cell cycle control through downregulation of *AURKA* (78), impaired maintenance of barrier integrity through downregulation of *SPINK4* (79), reduced glucuronidation of drugs and other compounds through downregulation of *UTG1A8* (80), compromised cholesterol homeostasis and lipoprotein production through downregulation of *PCSK9* (81), and increased microbiome regulation through upregulation of lactoferrin (34). Despite the lack of scientific evidence directly linking methane with these mechanisms, its indirect effects on cellular pathways, such as apoptosis and inflammation, may explain some of these findings (82). Since methanogens usually coexist with H_2_ producers, any dysregulation in the microbiome that promotes methanogens’ growth will require a balanced host response, in this case, potentially performed by lactoferrin. In addition, lactoferrin, which sequestrates luminal iron and affects its availability to the gut microbiome (83), was downregulated in ISO, placing iron homeostasis as a common factor with possible opposite roles in H_2_S/CH_4_-associated conditions. Moreover, since ISO is associated with IBS (1), our data corroborate previous findings showing higher fecal lactoferrin levels in IBD vs IBS (84, 85). In contrast to ISO, SIBO did not show broad effects in gene expression in our cohort, but SIBO-only was associated with impaired cell proliferation via *TANGO6* downregulation (50), and inhibition of inflammatory genes through downregulation of *HAS3* (86). Finally, with the H_2_S-producer gavages we showed that important changes in H_2_S-regulated biological processes occurred, validating findings in ISO.

This study has limitations. Larger sample sizes may help to identify more robust gene expression changes especially in IMO and SIBO groups. Also, more evidence is needed to explain the impact of key microbes in IMO and SIBO on ISO findings (and vice versa), since, for instance, early-growth acceleration of *Fusobacterium nucleatum* and *E. coli* has previously been shown to be induced by *M. smithii* (87). Moreover, in the clinical context, understanding how to ideally treat SIBO and ISO will be important. It is also important to consider the possibility that there may be additional complexity to treating patients with both SIBO and ISO. Furthermore, a lack of DEGs in *D. piger* rats vs controls could be due to differences in ATCC vs clinical isolate strains' virulence, exposure duration, or large vs small intestine colonization preferences. Moreover, we could not classify the animals into groups according to their H_2_S breath levels as we did for the human subjects. Also, due to the exploratory nature of this work, further research is also necessary to validate our findings and determine the direct impacts of H_2_S and CH_4_ in ISO and IMO, respectively, and if redox imbalance and ferroptosis are a consequence of downregulation of *SLC7A11,* and dysregulation of other redox genes, in ISO. Lastly, analyzing small bowel microbiome profiles in ISO, IMO, and SIBO subjects via shotgun sequencing could help link microbial communities to host transcriptomics.

In conclusion, ISO was associated with a small bowel transcriptomic profile that differed from IMO and SIBO. ISO showed partial concordance with transcriptional changes in animals gavaged with known H_2_S producers, such as *F. varium* and *E. coli* K12. These findings support the concept that microbial H_2_S exposure contributes to modifications in mitochondrial function, redox balance, antioxidant response, immune signaling, epithelial fluid regulation, neuromuscular signaling, and transit-related pathways. The lack of shared DEGs across ISO-only, IMO-only, and SIBO-only comparisons further suggests that these are biologically distinct host-microbial states. In contrast to ISO, IMO and SIBO showed more limited transcriptomic effects in our cohort. Overall, these data identify ISO as an H_2_S-associated transcriptomic phenotype with relevance to diarrhea-predominant IBS mechanisms, while emphasizing the need for larger, mechanistic studies integrating small bowel microbiome profiling, breath tests, and host -omics to validate these pathways and clarify their therapeutic implications.

## MATERIALS AND METHODS

### Human subjects

A total of 85 subjects from the REIMAGINE (Revealing the Entire Intestinal Microbiota and its Associations with the Genetic, Immunologic, and Neuroendocrine Ecosystem) study (88) were selected for inclusion in the present analysis based on the availability of complete breath test results for CH_4_ and H_2_S, and of McConkey agar duodenal aspirate culture results for the determination of SIBO (88). Subject demographics, including age, sex, BMI, and race, are provided in File S1.

### Breath testing, duodenal aspirates, and duodenal tissue procurement and processing

In the REIMAGINE study, patients aged 18–85 years undergoing upper gastrointestinal endoscopy (EGD) or antegrade double balloon endoscopy without colonoscopy provide breath samples and duodenal aspirates and biopsies for analysis (88). For breath testing, single fasting end-expiratory breath samples are collected in aluminum-lined Mylar collection bags designed to preserve H_2_S and prevent gas escaping, and used for subsequent CH_4_ and H_2_S measurements by gas chromatography (Gemelli Biotech, Raleigh, NC, USA). Portions of the duodenal aspirates are plated on McConkey agar for the determination of SIBO, and duodenal biopsies are also stored in Allprotect prior to RNA extraction.

### Human study groups

Levels of CH_4_ and H_2_S on exhaled breath were used to determine IMO and ISO, respectively. IMO was determined when CH_4_ ≥ 10 ppm, and ISO was considered when H_2_S ≥ 1.5 ppm, but subjects were divided into 1.5–1.9 ppm and ≥2.0 ppm subgroups. Duodenal aspirate cultures on MacConkey agar were used to determine SIBO (≥10^3^ CFU/mL), as described previously (88). Breath test and culture results divided the groups into (i) ISO (with or without SIBO/IMO); (ii) non-ISO (with or without SIBO/IMO); (iii) ISO only (SIBO−, IMO−, ISO+ 1.5 or 2.0); (iv) SIBO (with or without IMO/ISO); (v) non-SIBO (with or without IMO/ISO); (vi) SIBO only (SIBO+, IMO−, ISO−); (vii) IMO (with or without SIBO/ISO); (viii) non-IMO (with or without SIBO/ISO); (ix) IMO only (IMO+, SIBO−, ISO−); and (x) NNN (SIBO−, IMO−, ISO−).

### Animal studies

For the animal studies, a total of 70 adult male Sprague Dawley rats (Inotiv, West Lafayette, IN) were used. All animals were housed in 12/12 light-dark cycles and had *ad libitum* access to food and water. The first rat cohort was described in Villanueva-Millan et al. (2). Briefly, 10-week-old male Sprague-Dawley rats were fed a chow diet and gavaged 3× with either 1× PBS (1 mL, *n* = 8), or 10^8^ CFU/mL of *D. piger* (ATCC29098, *n* = 16), or 10^8^ CFU/mL of *F. varium* (clinical isolate, *n* = 16), on days 0, 2, and 4. Animals were euthanized on day 21, and ~10 mg of ileal tissue was collected and stored in AllProtect (Qiagen). The second cohort of 10-week-old male Sprague-Dawley rats was gavaged daily with 10^8^ CFU/mL *E. coli* K12 (ATCC10798, *n* = 10), a facultative H_2_S producer, 1 mL PBS (*n* = 10), or 10^8^ CFU/mL *E. coli* BL21 (ATCCBAA-1025, *n* = 10), for 15 days. Ileal tissue samples were collected at euthanasia on day 16.

### Bacterial gavages and *in vitro* H_2_S production

The *D. piger* and *F. varium* gavages were described in Villanueva-Millan et al. (2). *E. coli* K12 and *E. coli* BL21 were mixed in 6 mL of Luria Bertoni (LB) broth. The tube containing LB broth and bacteria (Corning, Tewksbury, MA, USA) was loosely closed and aerobically incubated at 37°C. When growth was detected by the change in MacFarland turbidity in a Den-1 densitometer (GrantBio), 8 serial 1/10 dilutions were prepared in PBS (100 µL + 900 µL) and plated in LB agar plates (100 µL per plate). Plates were incubated at 37°C overnight. Single colonies were selected from each strain and placed in LB broth, vortexed, and incubated at 37°C. When the turbidity corresponding to 10^8^ CFU/mL was reached after 2–4 h, the liquid cultures were centrifuged at 4°C for 10 min at 3,000 × *g*. The supernatants were discarded, and the bacterial pellets were washed twice with PBS at 4°C for 10 min at 3,000 × *g*, resuspended in PBS, and used in the gavages. Liquid bacterial cultures from *E. coli* BL21 and *E. coli* K12 were plated on *Desulfovibrio* agar according to the previously published protocol (89) to confirm H_2_S production after 72 h of anaerobic growth, as indicated by the presence of a clear black halo due to iron sulfide (FeS) formation (89).

### Total RNA isolation and quantification

Total RNA was isolated from human duodenal biopsies and rat ileal samples using the RNeasy Plus Mini Kit (Qiagen, Hilden, Germany) protocol with some modifications for the optimal isolation of short and long RNAs and removal of all genomic DNA (gDNA). First, ~10 mg of tissue was added to a 1.5 mL tube containing a 5 mm bead (Qiagen, Hilden, Germany) and lysed in 600 µL of RLT buffer for 4 min at 30 Hz in a TissueLyzer (Qiagen, Hilden, Germany). gDNA was removed using a gDNA Eliminator spin column. The flow-through was mixed with 1.5 volumes of 100% ethanol, and 700 µL of this mix was added to a RNeasy Mini spin column placed in a 2 mL collection tube. After centrifugation at full speed for 15 s, the flow-through was discarded, and the remaining mix was added to the same RNeasy Mini spin column for additional centrifugation. The flow-through was discarded again, and the column was washed twice with 500 µL of buffer RPE at full speed, first for 2 min, then for 15 s. The RNeasy Mini spin column was placed in a new collection tube and centrifuged for 1 min at full speed to dry the column. The last step consisted of adding 40 µL of RNeasy-free water to the column for elution of the total RNA containing miRNA, and centrifugation at full speed for 1 min. This first eluate underwent a second and identical RNA isolation to prevent DNA contamination by adding 600 µL of RLT plus to the 40 µL tube and following the protocol described above. The second RNA eluate was considered fully isolated and gDNA-free. Total RNA quantification and purity were obtained on a Nanodrop One (Thermo Scientific, Waltham, MA, USA). The RNA integrity number was determined on a 2100 Bioanalyzer using the RNA 6000 nano kit and RNA chip (Agilent, Santa Clara, CA, USA).

### mRNA library preparation and sequencing

Libraries were prepared with 500 ng RNA following the TruSeq Stranded mRNA protocol (Illumina, San Diego, CA, USA). Traces of adapter dimers, which were identified by peaks between the 100 and 150 bp region in the 2100 Bioanalyzer, were removed with an additional clean-up step using AMPure XP beads (Beckman Coulter, Brea, CA, USA). Final libraries underwent quality control checks on a 2100 Bioanalyzer using DNA 1000 Kits (Agilent, Santa Clara, CA, USA), and quantified in duplicate using Qubit 1x dsDNA BR Kits in Qubit 4 (Invitrogen). All human libraries were diluted to 2 nM, pooled accordingly, and denatured following the NovaSeq X Plus Denature and Dilute Libraries standard protocol (Illumina) for human samples, with a final load concentration of 120–130 pM per run. For *E. coli*-gavaged rats (and respective controls), all libraries were diluted to 1 nM, pooled, and denatured following the NextSeq System Denature and Dilute Libraries Guide (Illumina) using the Standard Normalization Method for High Output Kits, with a final load concentration of 1.75 pM, and sequenced on a NovaSeq 6000 (Illumina). All libraries from *D. piger*- and *F. varium*-gavaged rats (and respective controls) followed the same pooling and denaturing steps for *E. coli*-gavaged rats, but the loading concentration was 1.6 pM on a NextSeq 550 (Illumina). Animal and human sequencing runs had 200 cycles, except for the *E. coli*-gavaged rat samples (and controls) which were sequenced using a 150-cycle cartridge (Illumina). Batch effects were controlled by using internal controls across different runs for the human samples and for the *F. varium* and *D. piger* vs the control cohort. All samples in the *E. coli* cohorts were sequenced in a single run, so an internal control was necessary. After sequencing completion, fastq files were obtained from Basespace (Illumina) and uploaded into the CLC Genomics Workbench (Qiagen) for differential gene expression analysis. Human and animal data were analyzed in CLC v.25. The RNA-Seq Analysis tool was used to annotate genes and transcripts using the *Homo sapiens* hg38 or *Rattus norvegicus* GRCr8 as references for genes and transcripts from humans or rats, respectively. The mapping settings were default, with 10 as the maximum number of hits for a read. The expression settings were set for reverse strand, bulk sequencing, and total counts expression value, and a minimum read count fusion gene table of 5. Expression track files at the gene level were obtained and used for the analysis of differential expression between groups using the differential expression for RNA-Seq tool after applying a counts per million (CPM) threshold. Genes that did not obtain the CPM thresholds were filtered out in Excel, and total counts from the remaining genes were imported back to CLC for differential expression. The RNAseq analysis tool uses multi-factorial statistics based on a negative binomial generalized linear model, and trimmed mean of M-values as the normalization method. CPM values were used in comparisons. All groups were compared against the control group (differential expression in two groups). Differentially expressed mRNAs (identified in the text as DEGs) were annotated using their respective gene symbols.

### Functional enrichment analysis

Top Gene Ontology (GO) cellular processes and cellular components were obtained from ShinyGO v 0.80 (90–92) using DEG symbols as the input list, and significant pathways had *q* <0.05 (93). DEG symbols, DEG Ensembl IDs, and DEG log_2_ fold changes were used on IPA to predict activation or inhibition of significant pathways (FDR < 0.05) by performing core analysis.

### Quantification and statistical analyses

For human data, due to the complexity of cellular composition of small intestinal samples, and to prioritize sensitivity in identifying potential biomarkers in a heterogeneous population, genes were considered differentially expressed when the FDR *P* value (*q*-value, reported here as *q*) was <0.1 and the |fold change| (FC) was ≥1.3 (|Log_2_FC| ≥ 0.38), with CPM >0.85 in at least 50% of samples in a group. For animal data, genes were considered differentially expressed when *q* < 0.05, and |FC| ≥ 1.3 (|Log_2_FC| ≥ 0.38), with CPM thresholds respecting a minimum of 10 mapped reads in at least 50% of samples in a group. MetaboAnalyst v.5.0 (https://www.metaboanalyst.ca/) was used to obtain principal component analysis (PCA) using log_2_ (CPM + 1) transformed gene expression data; no additional filter was applied for data upload, 95% confidence intervals were distinguished by using ellipses, and a PERMANOVA test based on 999 permutations was performed to examine the differences in group centroids. Venn diagrams were designed on the Bioinformatics and Evolutionary Genomics website (https://bioinformatics.psb.ugent.be/webtools/Venn/) and using EVenn (https://www.bic.ac.cn/EVenn/#/) (94). Continuous variables, such as age and BMI, were compared by *t*-test (normal distribution) or the Mann-Whitney test on GraphPad Prism v9.2.0, and categorical variables were compared by Fisher’s exact test (two-tailed; 2 × 2) or Pearson chi-square test on SPSS v.23. Volcano plots were obtained from GraphPad Prism v9.2.0.

## Data Availability

The data sets generated during this study are available at the National Center for Biotechnology Information (NCBI) BioProject Repository (https://www.ncbi.nlm.nih.gov/bioproject) under BioProject ID PRJNA1285414 (animals) and PRJNA1285966 (humans).

## SUPPLEMENTAL MATERIAL

Figs S1-S6 msystems.00458-26-s0001.pdf Fig. S1 to S6.

Tables S1-S2 msystems.00458-26-s0002.pdf Tables S1 and S2.

File S1 msystems.00458-26-s0003.xlsx Metadata for human samples.

File S2 msystems.00458-26-s0004.xlsx Differentially expressed genes identified in each comparison for the human samples (REIMAGINE cohort).

File S3 msystems.00458-26-s0005.xlsx Percentage of reads mapped to the human and rat reference genomes.

File S4 msystems.00458-26-s0006.xlsx DEGs in rat groups.

ASM does not own the copyrights to Supplemental Material that may be linked to, or accessed through, an article. The authors have granted ASM a non-exclusive, world-wide license to publish the Supplemental Material files. Please contact the corresponding author directly for reuse.
