# Supplementary material for: Transcriptomics profiles in intestinal sulfide overproduction, small intestinal bacterial overgrowth, and intestinal methanogen overgrowth
Source: mSystems. 2026 Jun 29;11(7):e00458-26. doi: 10.1128/msystems.00458-26 (PMC13386965; doi:10.1128/msystems.00458-26)
Supplement: Figs S1-S6 — Fig. S1 to S6. [file msystems.00458-26-s0001.pdf]

Supplemental Information

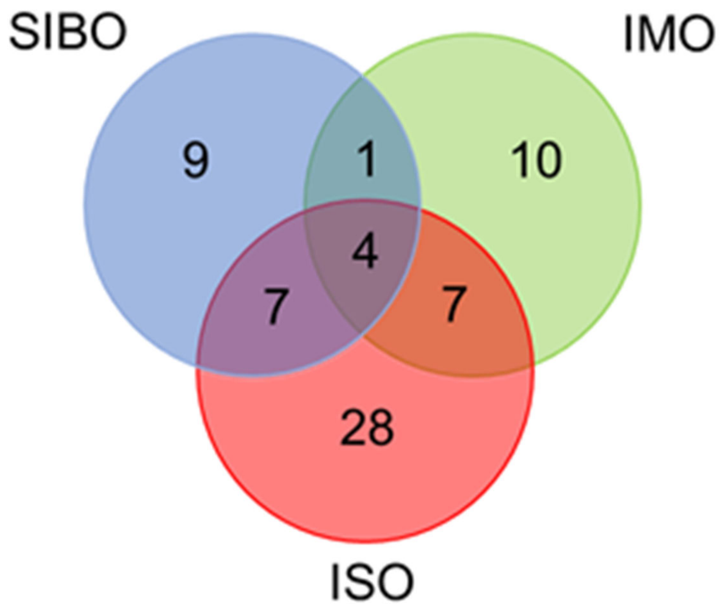

Figure S1. Intestinal methanogen overgrowth (IMO), intestinal sulfide overproduction (ISO) and small intestinal bacterial overgrowth (SIBO) distribution in the participants of the study.

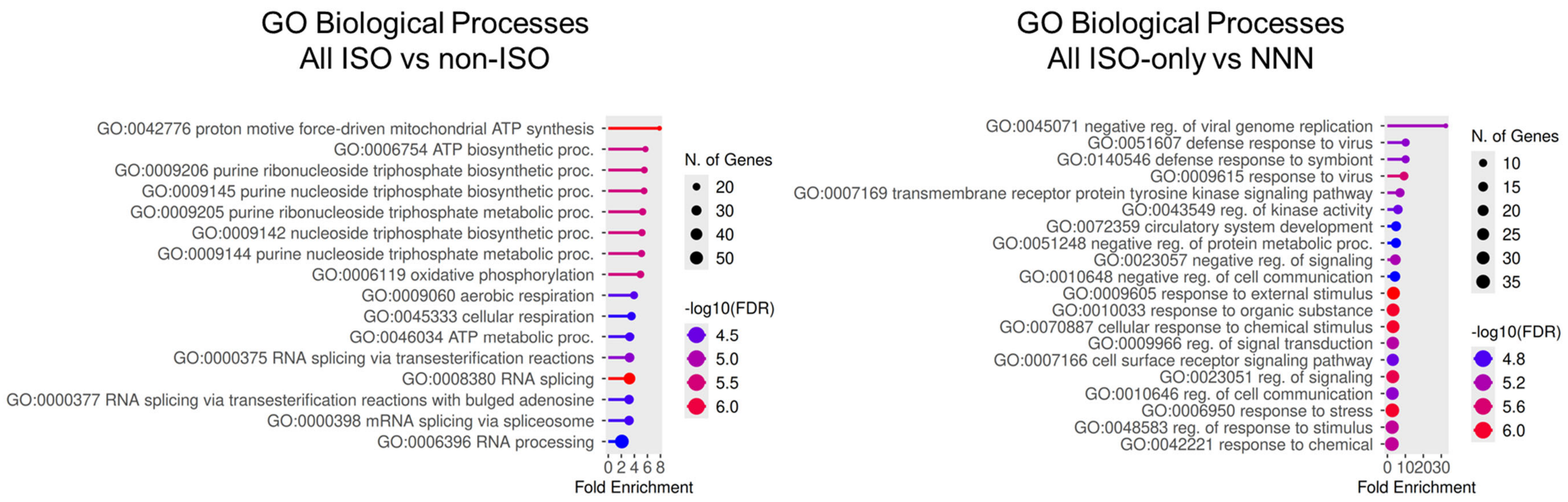

Figure S2. Biological Processes associated with the differentially expressed genes in ISO comparisons when FDR <0.05. GO, Gene ontology; ISO, Intestinal sulfide overproduction; NNN, non-ISO, non-IMO, non-SIBO.

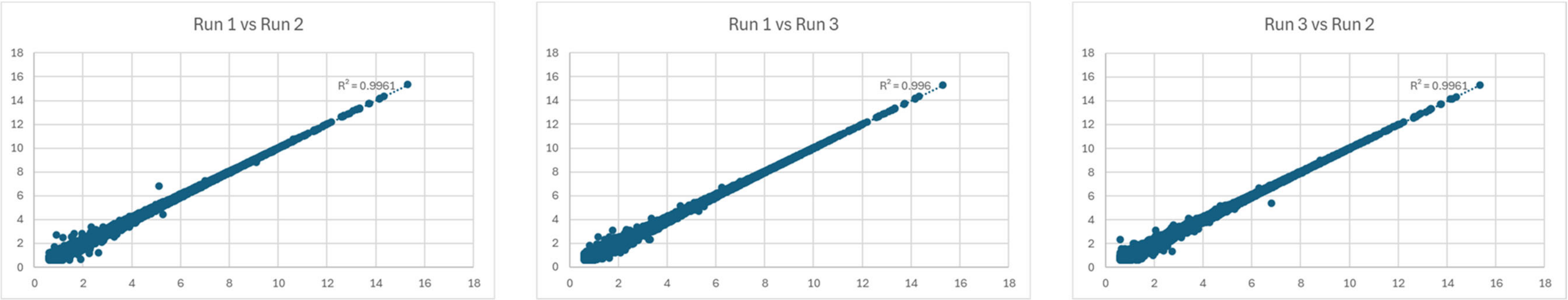

Figure S3. Animal model for gavages with *Desulfovibrio piger* and *Fusobacterium varium*. Rats were gavaged 3 times with either 1mL of PBS or 10^8 CFU/mL of *Desulfovibrio piger* or *Fusobacterium varium and* were euthanized 17 days after. Stool samples were collected one day before euthanasia for stool H_2S measurements, and small bowel was collected during euthanasia.

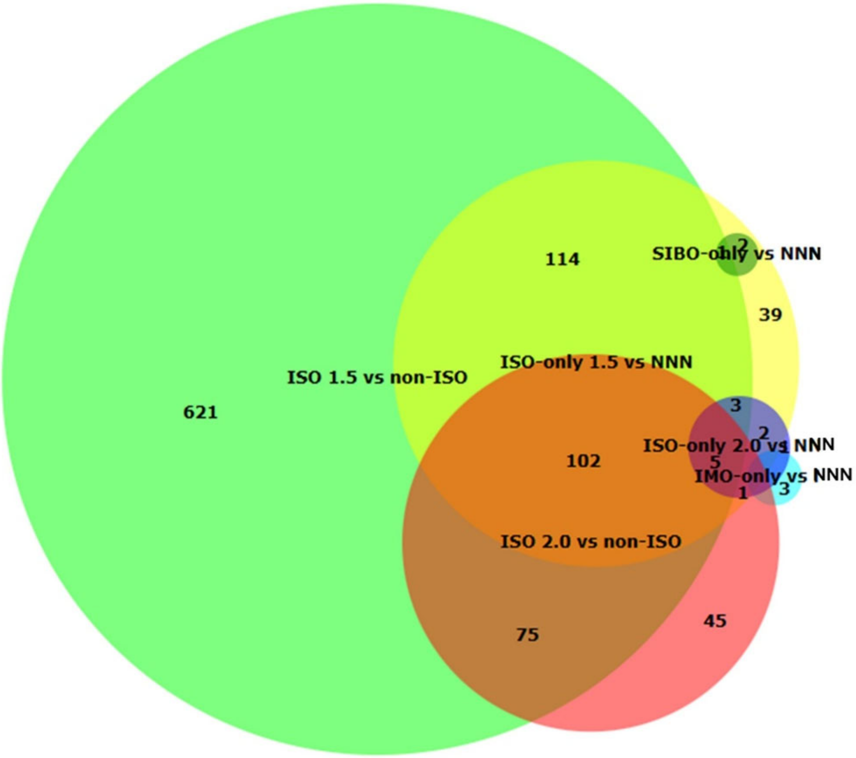

Figure S4. Venn diagram of the differentially expressed genes (DEGs) in all human group comparisons.

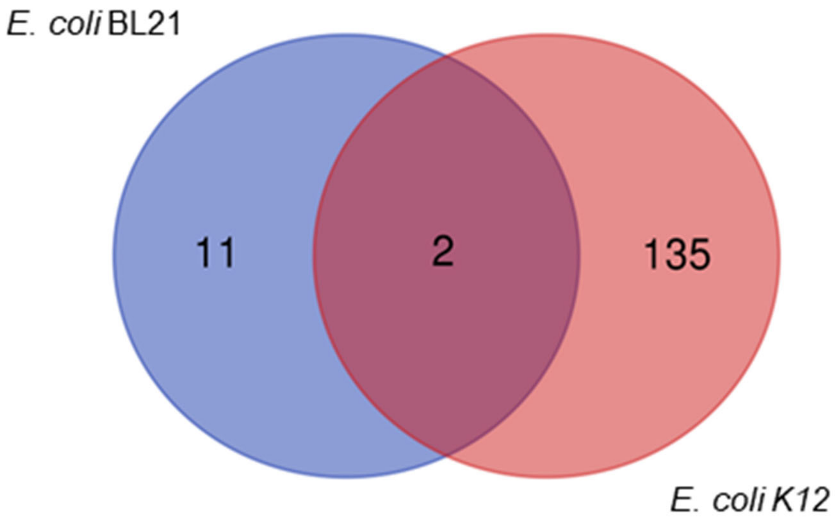

Figure S5. Venn diagram of the differentially expressed genes (DEGs) in *E. coli* K12 vs *E. coli* BL21. DEGs were FDR<0.05 and $|Log_2FC| \geq 0.38$.

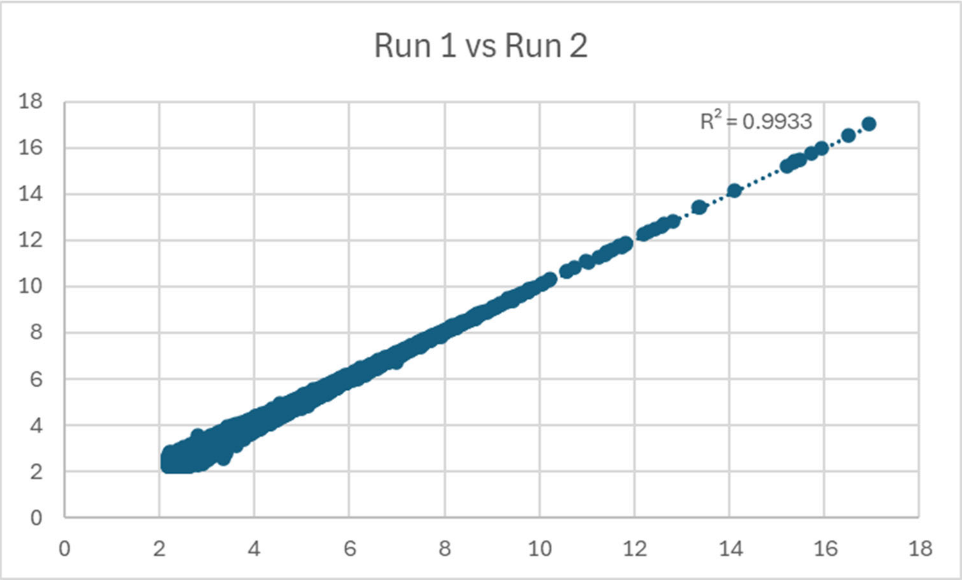

Figure S6. Correlation plot of the internal control in RNA sequencing from *D. piger* and *F. varium* vs control animal cohort. Log $_2$ (CPM+1) values were used for the plots. Low expressed genes (CPM <3.5 in both samples) were removed from the analysis.
