# Supplementary material for: Transcriptomics profiles in intestinal sulfide overproduction, small intestinal bacterial overgrowth, and intestinal methanogen overgrowth
Source: mSystems. 2026 Jun 29;11(7):e00458-26. doi: 10.1128/msystems.00458-26 (PMC13386965; doi:10.1128/msystems.00458-26)
Supplement: Tables S1-S2 — Tables S1 and S2. [file msystems.00458-26-s0002.pdf]

Table S1. Demographics in SIBO only, IMO only, and ISO only vs NNN

	McConkey agar culture (n=28)			Fasted breath test (n=57)								
	NNN (n=19)	SIBO-only (n=9)	*P* value	NNN (n=19)	IMO-only (n=10)	*P* value	NNN (n=19)	ISO only $H_2S \geq 2.0$ppm (n=12)	*P* value	NNN (n=19)	ISO-only $H_2S \geq 1.5$ppm (n=16)	*P* value
Age (years)	56±13	64±15	0.076	56±13	59±14	0.456	56±13	59±13	0.846	56±13	60±14	0.441
Female	13 (68%)	7 (78%)	1.000	13 (68%)	12 (55%)	0.804	13 (68%)	5 (42%)	0.262	13 (68%)	8 (50%)	0.317
BMI (kg/m^2)	27±8	24±4	0.498	27±8	25±5	0.247	27±8	27±7	0.586	27±8	26±4	0.987
Caucasian	17 (90%)	7 (78%)	0.331	17 (90%)	9 (90%)	0.691	17 (90%)	8 (67%)	0.246	17 (90%)	15 (94%)	0.646

Characteristics are presented as either mean ± standard deviation or total counts (percentage). Fisher exact test was performed in 2x2 categorical variables comparison on SPSS v.24, otherwise, Pearson chi-square was considered. Numeric variables were compared using either t-test (normal distribution) or Mann-Whitney on GraphPad Prism v.9.5.1. BMI, Body mass index.

Table S2. Top 10 indications for endoscopy in the study cohort

Category	Number of cases	%
GERD	9	10.6
Abdominal pain	9	10.6
Pancreas	8	9.4
Esophagus	8	9.4
Small bowel	4	4.7
Functional GI disorder	6	7.1
Dysphagia	6	7.1
Tumor	3	3.5
Gastric	5	5.9
Biliary disease	2	2.4
Other	25	29.4

GERD, Gastroesophageal reflux disease; GI, Gastrointestinal.
